# 9,10-Dibromo­phenanthrene

**DOI:** 10.1107/S1600536812042353

**Published:** 2012-10-20

**Authors:** Ruri Yokota, Chitoshi Kitamura, Takeshi Kawase

**Affiliations:** a Department of Materials Science and Chemistry, Graduate School of Engineering, University of Hyogo, 2167 Shosha, Himeji, Hyogo 671-2280, Japan

## Abstract

The mol­ecule of the title compound, C_14_H_8_Br_2_, is almost planar [maximum deviation 0.0355 (7) Å] and possesses crystallographic twofold (*C*2) symmetry. In the crystal, the mol­ecules form face-to-face slipped anti­parallel π–π stacking inter­actions along the *c* axis with an inter­planar distance 3.471 (7) Å, centroid–centroid distances of 3.617 (5)–3.803 (6) Å.

## Related literature

For the first synthesis of the title compound, see: Schmidt & Ladner (1904 ▶). For the synthesis of 2,2′-bis­(dibromo­meth­yl)biphenyl, see: Bacon & Bankhead (1963 ▶). For a related structure, see: Yokota *et al.* (2012 ▶).
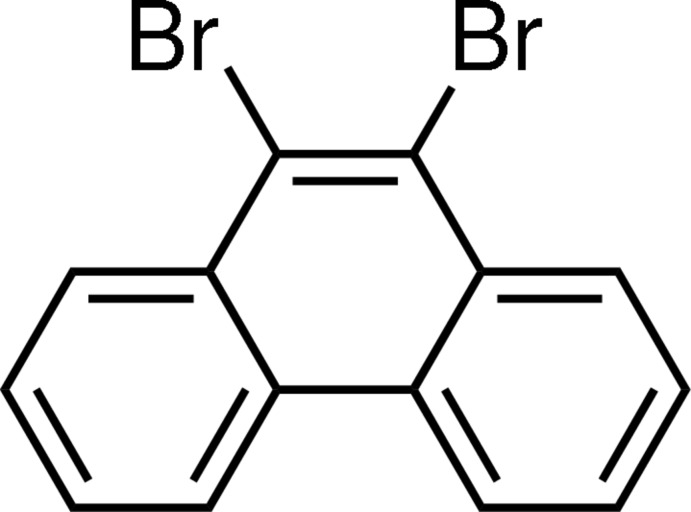


## Experimental

### 

#### Crystal data

C_14_H_8_Br_2_
*M* *_r_* = 336.02
Monoclinic, 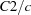

*a* = 18.2630 (15) Å
*b* = 9.0963 (8) Å
*c* = 7.3025 (6) Å
β = 114.499 (2)°
*V* = 1103.91 (16) Å^3^
*Z* = 4
Mo *K*α radiation
μ = 7.31 mm^−1^
*T* = 223 K
0.5 × 0.1 × 0.08 mm

#### Data collection

Rigaku R-AXIS RAPID diffractometer
Absorption correction: numerical (*NUMABS*; Higashi, 1999 ▶) *T*_min_ = 0.160, *T*_max_ = 0.558
5151 measured reflections
1257 independent reflections
1011 reflections with *I* > 2σ(*I*)
*R*_int_ = 0.028

#### Refinement

*R*[*F* ^2^ > 2σ(*F* ^2^)] = 0.033
*wR*(*F* ^2^) = 0.116
*S* = 1.25
1257 reflections
73 parameters
H-atom parameters constrained
Δρ_max_ = 0.67 e Å^−3^
Δρ_min_ = −1.49 e Å^−3^

### 

Data collection: *RAPID-AUTO* (Rigaku, 1999 ▶); cell refinement: *PROCESS-AUTO* (Rigaku, 1998 ▶); data reduction: *PROCESS-AUTO*; program(s) used to solve structure: *SIR2004* (Burla *et al.*, 2005 ▶); program(s) used to refine structure: *SHELXL97* (Sheldrick, 2008 ▶); molecular graphics: *ORTEP-3 for Windows* (Farrugia, 1997 ▶); software used to prepare material for publication: *WinGX* (Farrugia, 1999 ▶).

## Supplementary Material

Crystal structure: contains datablock(s) global, I. DOI: 10.1107/S1600536812042353/rz5013sup1.cif

Structure factors: contains datablock(s) I. DOI: 10.1107/S1600536812042353/rz5013Isup2.hkl

Supplementary material file. DOI: 10.1107/S1600536812042353/rz5013Isup3.cml

Additional supplementary materials:  crystallographic information; 3D view; checkCIF report

## Figures and Tables

**Fig. 1.**
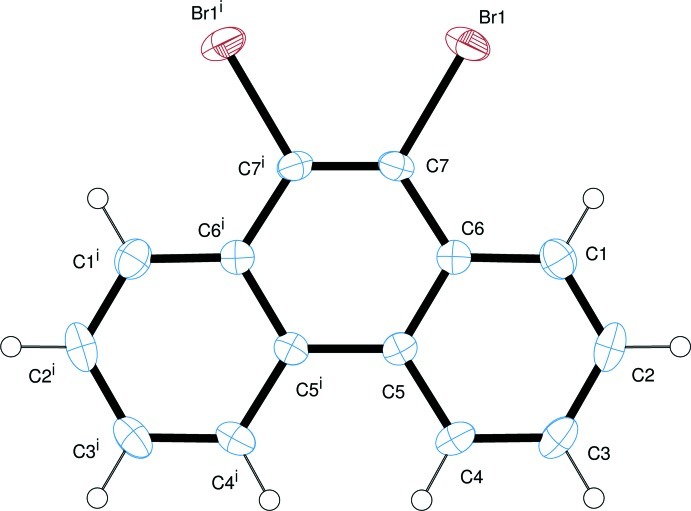
The molecular structure of the title compound, showing 40% probability displacement ellipsoids. Symmetry code: (i) -x + 1, y, -z + 1/2.

**Fig. 2.**
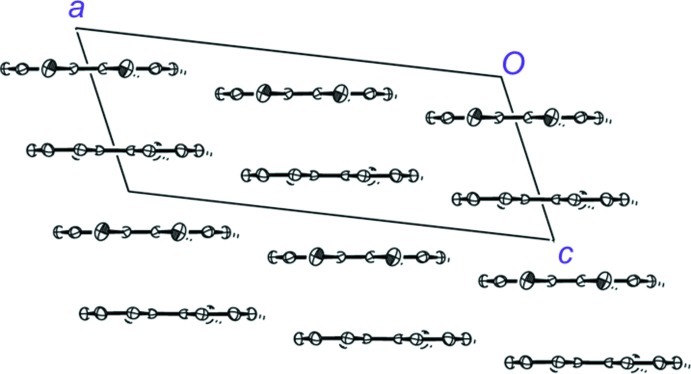
Packing diagram of the title compound viewed along the b axis. Hydrogen atoms are omitted for clarity.

## supplementary crystallographic information

### Comment

Phenanthrene is a polycylic aromatic hydrocarbon (PAH) as well as an important starting material toward fuctionalized π-conjugated molecules. The title compound, 9,10-dibromophenanthrene, was first prepared by Schmidt & Ladner (1904). However, the method involved relatively vigorous reaction conditions, and the title compound was not easily accessible. Therefore, the development of milder methods was pursued. Recently, we established a new method for the preparation of the title compound. Thus, treatment of 2,2'-bis(dibromomethyl)biphenyl (Bacon & Bankhead, 1963) with potassium *t*-butoxide yielded the title compound in a high yield. Securement of the title compound let to obtain single crystals suitable for X-ray analysis. We report herein the crystal structure of the title compound.

The molecular structure of the title compound is shown in Fig. 1. The molecule possesses *C*_2_ symmetry, and half of the formula unit is crystallographically independent. The molecule is almost planar with the maximum deviation of 0.0355 (7) Å for Br1. The bonds lengths and angles are in good agreement with the standard values. As shown in Fig. 2, the molecules form face-to-face slipped antiparrallel π-π stacking along the direction of the *c* axis. The interplanar distance is 3.471 (7) Å and controid–centroid distances of 3.617 (5)-3.803 (6) Å. Recently, we have reported the crystal structure of 3,6-dibromophenanthrene (Yokota *et al.*, 2012), whose feature was a herrinbone-like arrangement, indicating the difference in packing arrangement depending on the positions of bromo substituents.

### Experimental

2,2'-Bis(dibromomethyl)biphenyl, as a starting material, was prepared according to the method described by Bacon & Bankhead (1963). To an ice-cooled solution of 2,2'-bis(dibromomethyl)biphenyl (300 mg, 0.60 mmol) in DMF (6 ml), pottasium *t*-butoxide (1.00 g, 9.05 mmol) was added. After stirring for 30 min, the reaction was quenched with 6*M* HCl. The resulting solid was extracted with toluene, washed with brine, and dried over Na_2_SO_4_. After evaporation, column chromatography on silica gel (hexane-CH_2_Cl_2_) produced the title compound (161 mg, 79%) as a pale yellow solid. Single crystals suitable for X-ray analysis were obtained by slow evaporation from a toluene solution.

### Refinement

All the aromatic H atoms were positioned geometrically and refined using a riding model with C—H = 0.94 Å and *U*_iso_(H) = 1.2*U*_eq_(C).

### Crystal data

**Table:**

C_14_H_8_Br_2_	*F*(000) = 648
*M**_r_* = 336.02	*D*_x_ = 2.022 Mg m^−^^3^
Monoclinic, *C*2/*c*	Mo *K*α radiation, λ = 0.71073 Å
Hall symbol: -C 2yc	Cell parameters from 3170 reflections
*a* = 18.2630 (15) Å	θ = 3.0–27.5°
*b* = 9.0963 (8) Å	µ = 7.31 mm^−^^1^
*c* = 7.3025 (6) Å	*T* = 223 K
β = 114.499 (2)°	Needle, colourless
*V* = 1103.91 (16) Å^3^	0.5 × 0.1 × 0.08 mm
*Z* = 4	

### Data collection

**Table:**

Rigaku R-AXIS RAPID diffractometer	1257 independent reflections
Radiation source: fine-focus sealed x-ray tube	1011 reflections with *I* > 2σ(*I*)
Graphite monochromator	*R*_int_ = 0.028
Detector resolution: 10 pixels mm^-1^	θ_max_ = 27.5°, θ_min_ = 3.6°
ω scans	*h* = −23→23
Absorption correction: numerical (*NUMABS*; Higashi, 1999)	*k* = −11→11
*T*_min_ = 0.160, *T*_max_ = 0.558	*l* = −9→9
5151 measured reflections	

### Refinement

**Table:**

Refinement on *F*^2^	Primary atom site location: structure-invariant direct methods
Least-squares matrix: full	Hydrogen site location: inferred from neighbouring sites
*R*[*F*^2^ > 2σ(*F*^2^)] = 0.033	H-atom parameters constrained
*wR*(*F*^2^) = 0.116	*w* = 1/[σ^2^(*F*_o_^2^) + (0.0313*P*)^2^ + 8.7358*P*] where *P* = (*F*_o_^2^ + 2*F*_c_^2^)/3
*S* = 1.25	(Δ/σ)_max_ < 0.001
1257 reflections	Δρ_max_ = 0.67 e Å^−^^3^
73 parameters	Δρ_min_ = −1.49 e Å^−^^3^
0 restraints	

### Special details

**Table:**

Geometry. All s.u.'s (except the s.u. in the dihedral angle between two l.s. planes) are estimated using the full covariance matrix. The cell s.u.'s are taken into account individually in the estimation of s.u.'s in distances, angles and torsion angles; correlations between s.u.'s in cell parameters are only used when they are defined by crystal symmetry. An approximate (isotropic) treatment of cell s.u.'s is used for estimating s.u.'s involving l.s. planes.
Refinement. Refinement of *F*^2^ against ALL reflections. The weighted *R*-factor *wR* and goodness of fit *S* are based on *F*^2^, conventional *R*-factors *R* are based on *F*, with *F* set to zero for negative *F*^2^. The threshold expression of *F*^2^ > 2σ(*F*^2^) is used only for calculating *R*-factors(gt) *etc*. and is not relevant to the choice of reflections for refinement. *R*-factors based on *F*^2^ are statistically about twice as large as those based on *F*, and *R*- factors based on ALL data will be even larger.

### Fractional atomic coordinates and isotropic or equivalent isotropic displacement parameters (Å^2^)

**Table:**

	*x*	*y*	*z*	*U*_iso_*/*U*_eq_	
C1	0.3372 (3)	0.4275 (6)	0.2001 (7)	0.0341 (11)	
H1	0.3099	0.3382	0.1903	0.041*	
C2	0.2976 (3)	0.5571 (7)	0.1900 (8)	0.0395 (12)	
H2	0.2438	0.5564	0.1738	0.047*	
C3	0.3375 (3)	0.6900 (6)	0.2037 (8)	0.0388 (12)	
H3	0.3103	0.7791	0.1962	0.047*	
C4	0.4163 (3)	0.6916 (5)	0.2284 (7)	0.0320 (10)	
H4	0.4425	0.7822	0.2387	0.038*	
C5	0.4586 (3)	0.5595 (5)	0.2384 (6)	0.0248 (9)	
C6	0.4168 (3)	0.4248 (5)	0.2245 (6)	0.0257 (9)	
C7	0.4612 (3)	0.2907 (5)	0.2373 (7)	0.0268 (9)	
Br1	0.40654 (4)	0.11098 (6)	0.22296 (10)	0.0491 (2)	

### Atomic displacement parameters (Å^2^)

**Table:**

	*U*^11^	*U*^22^	*U*^33^	*U*^12^	*U*^13^	*U*^23^
C1	0.034 (2)	0.039 (3)	0.031 (2)	−0.005 (2)	0.015 (2)	−0.001 (2)
C2	0.025 (2)	0.058 (3)	0.036 (3)	0.011 (2)	0.013 (2)	0.001 (2)
C3	0.033 (3)	0.042 (3)	0.037 (3)	0.014 (2)	0.010 (2)	0.001 (2)
C4	0.036 (2)	0.027 (2)	0.032 (2)	0.0076 (19)	0.012 (2)	−0.0008 (19)
C5	0.028 (2)	0.025 (2)	0.020 (2)	0.0015 (17)	0.0090 (18)	−0.0012 (16)
C6	0.027 (2)	0.028 (2)	0.020 (2)	0.0002 (17)	0.0083 (18)	0.0004 (17)
C7	0.033 (2)	0.021 (2)	0.029 (2)	−0.0036 (17)	0.015 (2)	−0.0008 (17)
Br1	0.0552 (4)	0.0270 (3)	0.0752 (5)	−0.0125 (2)	0.0373 (3)	−0.0035 (3)

### Geometric parameters (Å, º)

**Table:**

C1—C2	1.369 (7)	C4—C5	1.415 (6)
C1—C6	1.389 (6)	C4—H4	0.94
C1—H1	0.94	C5—C6	1.424 (6)
C2—C3	1.393 (8)	C5—C5^i^	1.449 (9)
C2—H2	0.94	C6—C7	1.447 (6)
C3—C4	1.375 (7)	C7—C7^i^	1.349 (9)
C3—H3	0.94	C7—Br1	1.896 (4)
			
C2—C1—C6	121.5 (5)	C5—C4—H4	119.4
C2—C1—H1	119.2	C4—C5—C6	117.5 (4)
C6—C1—H1	119.2	C4—C5—C5^i^	121.8 (3)
C1—C2—C3	119.7 (5)	C6—C5—C5^i^	120.7 (3)
C1—C2—H2	120.2	C1—C6—C5	119.7 (4)
C3—C2—H2	120.2	C1—C6—C7	123.5 (4)
C4—C3—C2	120.4 (5)	C5—C6—C7	116.8 (4)
C4—C3—H3	119.8	C7^i^—C7—C6	122.5 (2)
C2—C3—H3	119.8	C7^i^—C7—Br1	120.42 (14)
C3—C4—C5	121.2 (5)	C6—C7—Br1	117.1 (3)
C3—C4—H4	119.4		
			
C6—C1—C2—C3	0.2 (8)	C5^i^—C5—C6—C1	−179.3 (5)
C1—C2—C3—C4	−0.3 (8)	C4—C5—C6—C7	−179.3 (4)
C2—C3—C4—C5	0.6 (7)	C5^i^—C5—C6—C7	0.8 (7)
C3—C4—C5—C6	−0.8 (7)	C1—C6—C7—C7^i^	−179.4 (5)
C3—C4—C5—C5^i^	179.2 (5)	C5—C6—C7—C7^i^	0.5 (8)
C2—C1—C6—C5	−0.4 (7)	C1—C6—C7—Br1	−0.9 (6)
C2—C1—C6—C7	179.6 (5)	C5—C6—C7—Br1	179.1 (3)
C4—C5—C6—C1	0.6 (6)		

Symmetry code: (i) −*x*+1, *y*, −*z*+1/2.

## References

[bb1] Bacon, R. G. R. & Bankhead, R. (1963). *J. Chem. Soc.* pp. 839–845.

[bb2] Burla, M. C., Caliandro, R., Camalli, M., Carrozzini, B., Cascarano, G. L., De Caro, L., Giacovazzo, C., Polidori, G. & Spagna, R. (2005). *J. Appl. Cryst.* **38**, 381–388.

[bb3] Farrugia, L. J. (1997). *J. Appl. Cryst.* **30**, 565.

[bb4] Farrugia, L. J. (1999). *J. Appl. Cryst.* **32**, 837–838.

[bb5] Higashi, T. (1999). *NUMABS* Rigaku Corporation, Tokyo, Japan.

[bb6] Rigaku (1998). *PROCESS-AUTO* Rigaku Corporation, Tokyo, Japan.

[bb7] Rigaku (1999). *RAPID-AUTO* Rigaku Corporation, Tokyo, Japan.

[bb8] Schmidt, J. & Ladner, G. (1904). *Chem. Ber.* **37**, 4402–4405.

[bb9] Sheldrick, G. M. (2008). *Acta Cryst.* A**64**, 112–122. 10.1107/S0108767307043930 18156677

[bb10] Yokota, R., Kitamura, C. & Kawase, T. (2012). *Acta Cryst.* E**68**, o3100. 10.1107/S1600536812041621 PMC3515201 23284428

